# *Moraxella catarrhalis* Promotes Stable Polymicrobial Biofilms With the Major Otopathogens

**DOI:** 10.3389/fmicb.2019.03006

**Published:** 2020-01-15

**Authors:** Kirsten L. Bair, Anthony A. Campagnari

**Affiliations:** ^1^Department of Microbiology and Immunology, Jacobs School of Medicine and Biomedical Sciences, University at Buffalo, State University of New York, Buffalo, NY, United States; ^2^The Witebsky Center for Microbial Pathogenesis and Immunology, University at Buffalo, State University of New York, Buffalo, NY, United States

**Keywords:** otitis media, biofilm, polymicrobial, *Moraxella catarrhalis*, *Streptococcus pneumoniae*, non-typeable *Haemophilus influenzae*

## Abstract

Otitis media (OM) is a prevalent pediatric infection characterized by painful inflammation of the middle ear. The Gram-negative diplococcus *Moraxella catarrhalis* is a commensal of the nasopharynx and one of three leading causative agents of OM. The most recent work on this multifaceted disease indicates that biofilms and polymicrobial infections play a pivotal role in recurrent and chronic OM, which are difficult to eradicate using standard antibiotic protocols. Although there have been significant advances in OM research, the actual bacterial and viral interactions leading to pathogenesis remain largely uncharacterized. However, colonization and persistence in the nasopharynx is clearly an essential first step. In this study, we assessed the role *M. catarrhalis* plays in the co-colonization and persistence of the other major otopathogens, *Streptococcus pneumoniae* and non-typeable *Haemophilus influenzae* (NTHi). We characterized both monomicrobial and polymicrobial biofilms using an *in vitro* nasopharyngeal colonization model. Biofilm assays were designed to mimic the nasopharynx and bacterial persistence was quantified over time. NTHi showed a steady and significant decline in viability over 20–48 h when this organism was in a dual species biofilm with *S. pneumoniae*. However, when *M. catarrhalis* was present in the polymicrobial biofilm NTHi survived for 48 h at 10^7^ CFU per mL. In addition, an isogenic *M. catarrhalis* catalase-deficient mutant was also fully capable of protecting NTHi from the bactericidal activity of *S. pneumoniae* in a polymicrobial biofilm. Our results show that *M. catarrhalis* promotes a favorable environment for stable polymicrobial biofilms by enhancing the survival of NTHi in the presence of *S. pneumoniae*. These data suggest that colonization with *M. catarrhalis* promotes stable co-colonization with other otopathogens.

## Introduction

There are more than 700 million cases of acute otitis media (AOM) diagnosed globally each year, with 50% of affected children under 5 years of age (Monasta et al., 2012). *Moraxella catarrhalis*, non-typeable *Haemophilus influenzae* (NTHi) and *Streptococcus pneumoniae* cause approximately 95% of AOM cases creating an incredible economic burden on healthcare systems (Broides et al., 2009). In the United States, it is estimated that AOM is responsible for 4.3 billion dollars in health-related costs (Tong et al., 2018). In addition to being the most common reason for doctor’s office visits among children, AOM is also currently the most common reason for antibiotic use in the pediatric population. Recent studies have shown antibiotic resistance and decreased sensitivity developing among the major otopathogens (Pichichero, 2000a; Zielnik-Jurkiewicz and Bielicka, 2015; Sillanpaa et al., 2016; Korona-Glowniak et al., 2018). Further, the polymicrobial biofilms associated with AOM are incredibly resistant and difficult to treat using classic antibiotic protocols (Pichichero, 2000b; Leibovitz et al., 2003; Libson et al., 2005; Asher et al., 2008; Leibovitz, 2008; Korona-Glowniak et al., 2018). This is a result of conferred β-lactamase protection, quiescent bacteria within biofilms, poor antibiotic penetration and persister cells. When taken in combination with the continued prevalence of AOM in the post-vaccine era, these challenges demand novel preventative and treatment strategies.

Because all of these otopathogens can colonize asymptomatically, the interactions that occur in the nasopharynx that prevent or promote co-colonization play an important role in the steps that eventually lead to pathogenesis. Thus, we focused our studies on a more thorough evaluation of the possible events that occur during nasopharyngeal colonization. Providing a better understanding of the bacterial interactions that occur between the three primary otopathogens could lead to novel strategies for the prevention and treatment of AOM (Armbruster and Swords, 2010; Murphy, 2015).

To date, some of the dual species interactions of otopathogens have been characterized *in vitro* or *in vivo*. However, the ability to compare the three species in a single system has been limited by animal models that are less than ideal for the strict human pathogen *M. catarrhalis*. We have extended studies on these otopathogens by employing an *in vitro* nasopharyngeal colonization model adapted from previous studies originally designed for *S. pneumoniae* (Marks et al., 2012; Chao et al., 2017; Reddinger et al., 2018). The model mimics the conditions of the human nasopharynx including considerations for nasopharyngeal temperature, nutrient availability, aeration, and epithelial attachment.

Using this modified *in vitro* nasopharyngeal colonization model we assessed co-colonization dynamics of each otopathogen in dual species. Further, we analyzed interactions of all three otopathogens in triple species biofilms which have not been previously studied. Our results indicate that *M. catarrhalis* is able to promote survival of NTHi even in the presence of *S. pneumoniae* in triple species biofilms like those that have been previously shown to colonize the human nasopharynx (Hoa et al., 2009; Casey et al., 2010; Palmu et al., 2019).

## Materials and Methods

### Bacterial Strains and Culture Methods

*Moraxella catarrhalis* strain 7169 is a clinical middle ear isolate (Faden et al., 1997). Minimally passaged planktonic *M. catarrhalis* cultures were grown at 37°C, 180 RPM, aerobically in chemically defined pneumococcal growth media (CDM) as previously described (van de Rijn and Kessler, 1980). NTHi strain 86-028NP is a clinical isolate from a pediatric patient who underwent a tympanostomy for chronic otitis media (OM) (Kennedy et al., 2000). NTHi cultures were grow at 37°C, 180 RPM, aerobically in CDM. *S. pneumoniae* EF3030 is a serotype 19F OM isolate that was grown statically and anaerobically in CDM at 37°C (Andersson et al., 1983). The co-infection strains *M. catarrhalis* 11-01-125, *S. pneumoniae* 11-01-125 and NTHi 11-01-125 were isolated from the middle ear fluid of a pediatric patient during a polymicrobial infection and generously provided by Michael Pichichero, MD (Rochester, NY, United States). Each species was grown as outlined above. These isolates were maintained for long term storage at −80°C.

### Static Biofilms and Time Course Assay

**FIGURE 1:**
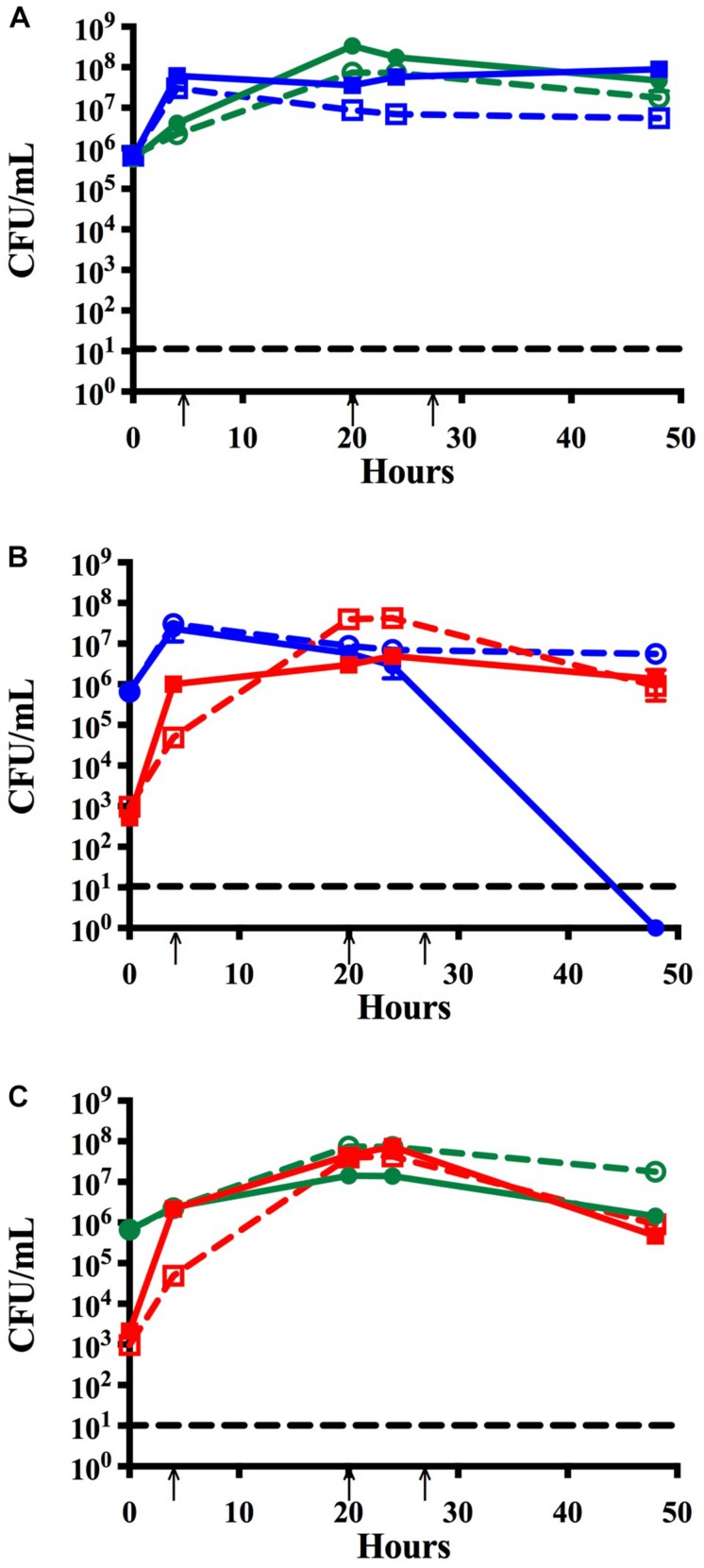
Time course assay analyzing monomicrobial and dual species biofilms *in vitro*. Bacteria were grown in monomicrobial (dashed) or dual species (solid) biofilms and maintained with media changes (black arrows) for 48 h. **(A)** NTHi86-028 NP (blue) and *M. catarrhalis* 7169 (green) monomicrobial and dual species biofilms. **(B)** NTHi86-028 NP (blue) and *S. pneumoniae* EF3030 (red) monomicrobial and dual species biofilms. **(C)** *M. catarrhalis* 7169 (green) and *S. pneumoniae* EF3030 (red) monomicrobial and dual species biofilms. Black dashed line indicates the limit of CFU detection. Each symbol and error bar represent the mean CFU per mL and standard deviation (SD) of biofilms at the respective time point. Data presented are from three independent assays with a minimum of two biologic replicates per assay.

**FIGURE 2:**
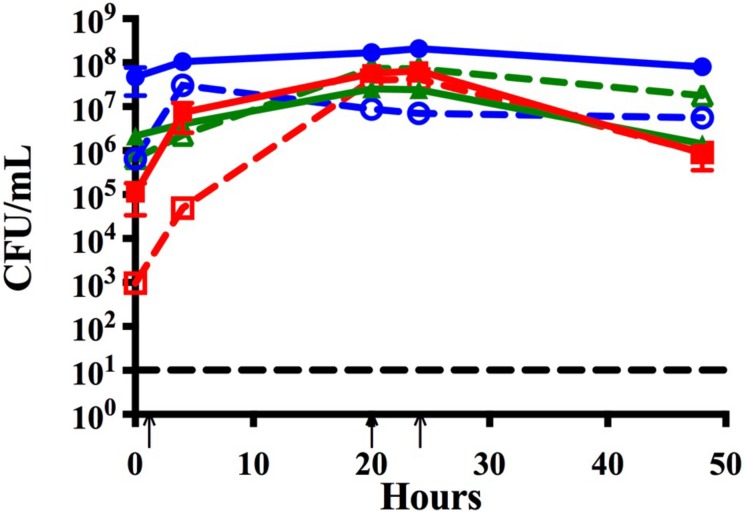
Time course assay analyzing monomicrobial and triple species biofilms *in vitro*. Bacteria were grown in monomicrobial (dashed) or triple species biofilms (solid). Biofilms were seeded simultaneously and maintained with media changes (black arrows) for 48 h. NTHi86-028 NP (blue), *M. catarrhalis* 7169 (green), and *S. pneumoniae* EF3030 (red) monomicrobial and triple species biofilms. Black dashed line indicates the limit of CFU detection. Each symbol and error bar represent the mean CFU per mL and standard deviation (SD) of biofilms at the respective time point. Data presented are from three independent assays with a minimum of two biologic replicates per assay.

**FIGURE 3:**
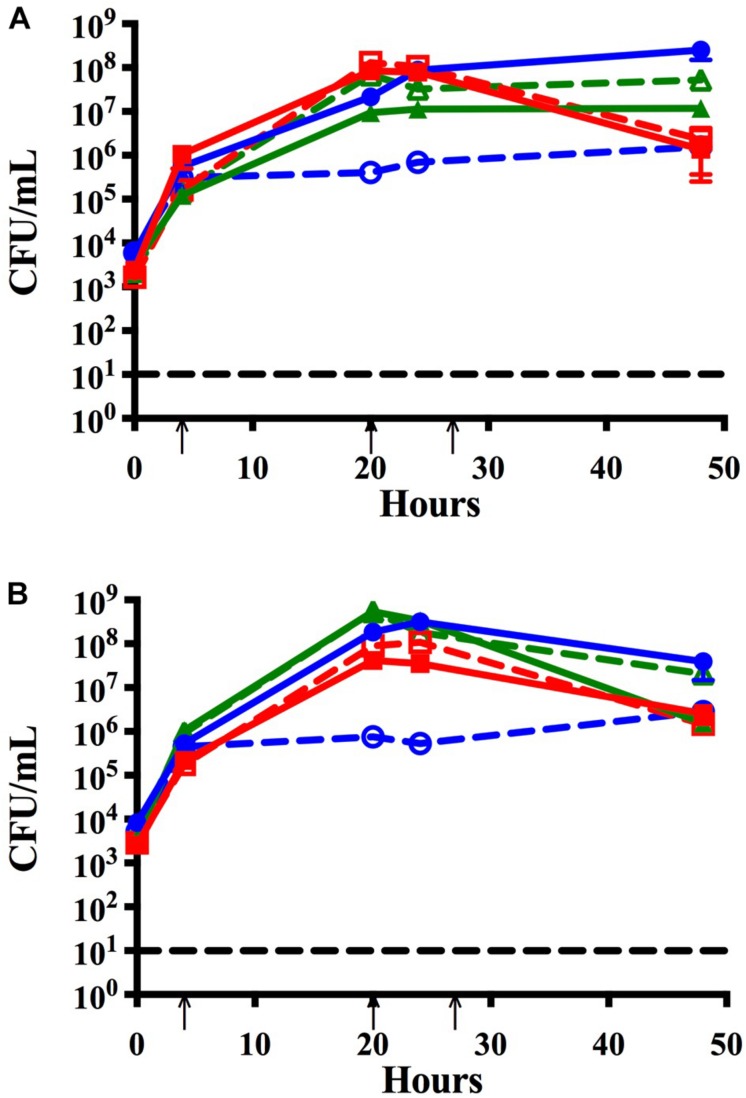
Time course assay to assess the effects of lower seeding concentrations and address strain specificity by analyzing trends of co-infection isolates. Bacteria seeded simultaneously at a lower concentration (∼10^5^ CFU per mL) and maintained with media changes (black arrows) for 48 h in monomicrobial (dashed) or triple species (solid) biofilms. Each symbol and error bar represent the mean CFU per mL and standard deviation (SD) of biofilms at the respective time point. Data presented are from three independent assays with a minimum of two biologic replicates per assay. Black dashed line indicates the limit of CFU detection. **(A)** NTHi86-028 NP (blue), *M. catarrhalis* 7169 (green), and *S. pneumoniae* EF3030 (red) monomicrobial and triple species biofilms. **(B)** NTHi 11-01-125 (blue), *M. catarrhalis* 11-01-125 (green) and *S. pneumoniae* 11-01-125 (red) monomicrobial and triple species biofilms.

Stationary *in vitro* biofilms were grown in 24-well plates on a monolayer of NCI-H292 bronchial carcinoma cells (ATCC CCL-1848) as previously described (van Schilfgaarde et al., 1995). Planktonic cultures were grown to an OD_600_ of ∼0.2 (∼10^7^ CFU per mL). *M. catarrhalis* and NTHi were used at this concentration whereas *S. pneumoniae* was diluted 1:100 prior to seeding (∼10^4^ CFU per mL). These inocula were used for the studies in Figures 1B,C, 2. For the data shown in Figure 3, all three otopathogens were used at a starting inoculum of ∼10^4^ CFU per mL. Wells received 350 μL of each culture during seeding of either monomicrobial, dual species or triple species biofilms and CDM was added to a final volume of 1050 μL. Biofilms were incubated statically at 34°C and 5% CO_2_. Media changes were completed at 4, 20, and 28 h post-seeding by replacing all spent media with 1 mL fresh CDM. Biofilm formation was quantified at 0, 4, 20, 24, and 48 h by carefully removing the supernatant and any planktonic or loosely attached bacteria and resuspending the biofilm in PBS via physical disruption with a pipette tip. Dilution plating onto selective media was utilized for CFU enumeration of *M. catarrhalis* (Mueller–Hinton agar + vancomycin at 3 mg per mL), *S. pneumoniae* (Blood agar + gentamicin at 4 mg per mL), and NTHi (Chocolate agar + clarithromycin at 2 mg per mL) at each time point. Plates were incubated for 24 h at 37°C and 5% CO_2_.

### Transwell Assay

Dual species *S. pneumoniae* EF3030 and NTHi 86-028NP biofilms were grown in 24-well plates on a fixed H292 cell monolayer. NTHi 86-028NP and *S. pneumoniae* EF3030 planktonic cultures were grown to an OD_600_ of ∼0.2 and diluted 1:100 for a seeding concentration of ∼10^5^ CFU per mL and 350 μL of the 1:100 culture dilution was used for seeding. A transwell insert was added to each well [0.4 μm PET cell-culture 24-well adapted insert (Corning Inc., Corning, NY, United States)]. Transwell inserts of the treated wells received 350 μL of *M. catarrhalis* 7169 prepared for seeding as discussed previously, and control wells were seeded with 350 μL CDM. Media changes were completed at 4, 20, and 28 h post-seeding by replacing the spent media in the well and in the transwell insert separately. At 24 and 48 h the spent media was removed and the inserts were placed into a sterile 24 well plate. The biofilms in the bottom of the 24-well plate and biofilms in the insert were each harvested in 1 mL PBS and dilution plated onto selective media for CFU enumeration as described above.

### Catalase Mutant Formation

The *M. catarrhalis* catalase mutant 7169ΔkatSpec1 was constructed in 7169 using the SOE-PCR technique to construct an insertion/deletion mutation (Thornton, 2016). In brief, amplicons containing 1350 nucleotides (nt) of genomic 7169 DNA upstream (using primers 2865/2868, Table 1) and 1364 nt downstream (using primers 2869/2864, Table 1) of *katA* were amplified using the non-A-tailing high-fidelity polymerase PFU (Agilent Technologies, Santa Clara, CA, United States). A third 895 nt amplicon was generated using primers 2870 and 2871 designed to flank the spectinomycin resistance cassette using pSpec as the template (Whitby et al., 1998; Plamondon et al., 2007). The 5′ region of primers 2868 and 2869 contained 20 nucleotides of homology to the primers 2870 and 2871 to allow for overlap and priming to give the final product containing the spectinomycin resistance cassette inserted within a 150 nt deletion internal to the *katA* coding region. This was accomplished by a final PCR reaction using primers 2864 and 2865 and the three purified amplicons Qiagen MinElute Reaction Cleanup Kit (Qiagen, Germantown, MD, United States). The resulting 3609 nt amplicon, containing the insertionally inactivated *katA*, was purified Qiagen MinElute Reaction Cleanup Kit and used to mutagenize 7169 by natural transformation, as described (Furano and Campagnari, 2003). Transformants were selected following overnight growth on Mueller–Hinton agar plates containing 15 μg per ml spectinomycin. Chromosomal DNA from transformant 7169ΔkatSpec1 was isolated and subjected to PCR analysis (with primers 2874 and 2875 designed to flank the site of homologous recombination) and sequence analysis confirmed the integration of the inactivated *katA* construct into the 7169 genome. We further confirmed the functional inactivation using a qualitative catalase test. A sterile inoculating needle was used to transfer a single colony into a droplet of 3% hydrogen peroxide. A negative test is indicated by the lack of O_2_ production, which is a product of hydrogen peroxide catalysis by catalase.

**TABLE 1:** Nucleotide sequences of oligonucleotide primers used for PCR-based mutagenesis.

**Primer**	**Sequence (5′–3′)**
2864	ACCGATGCCGAAATGGTCTT
2865	TTTGCTGCACAGTTTACCGC
2868	CTCCTCACTATTTTGATTAGACCTGATGAAACGCCTCTGG
2869	GAAAACAATAAACCCTTGCCCGATGCCGAAGCTGAAATG
2870	CCAGAGGCGTTTCATCAGGTCTAATCAAAATAGTGAGGAG
2871	CATTTCAGCTTCGGCATCGGGCAAGGGTTTATTGTTTTC
2874	AAACTTCCCACATGCAACGC
2875	CAACTAGAAGCACCGCCTCA

### Statistical Analyses

Student’s *t*-tests were used to compare the CFUs per mL of NTHi and *S. pneumoniae* exposed to *M. catarrhalis* or CDM in the filter well experiments. In addition, log transformed values of NTHi in the transwell assay (CDM treated, WT treated and catalase mutant treated) were checked for normal distribution using the Shapiro–Wilk test. Normally distributed data sets were analyzed by one-way ANOVA with Dunnett’s multiple comparison test. Alternatively, data sets that did not pass the Shapiro–Wilk test were analyzed using the non-parametric Kruskal–Wallis with Dunn’s multiple comparison test. All *P*-values were derived using a 95% confidence interval. The statistical analysis was performed with Prism 8 from GraphPad Software, Inc. (La Jolla, CA, United States).

## Results

### *Moraxella catarrhalis* Promotes the Growth of NTHi in Dual Species Biofilms *in vitro*

Previous studies have shown that colonization with *M. catarrhalis* occurs very early in life and *M. catarrhalis* is more likely to be present in polymicrobial AOM than infections resulting from a single bacterial species (Broides et al., 2009). Using our *in vitro* nasopharyngeal colonization model we assessed the relationship between *M. catarrhalis* and NTHi biofilms in physiologically relevant conditions. Biofilm formation was quantified over time via CFU enumeration. Figure 1A, shows that NTHi forms a slightly more robust biofilm, ∼1 log greater, in dual species biofilms with *M. catarrhalis* than in monomicrobial biofilms which is consistent with previously reported data (Mokrzan et al., 2018). This is a statistically significant increase in viable NTHi at 48 h as compared to NTHi grown in monomicrobial biofilms (Supplementary Figure S1A).

### NTHi Is Unable to Persist in Biofilms With *S. pneumoniae*

Although *S. pneumoniae* and NTHi each have a very high prevalence in OM individually, they are isolated together far less frequently (Casey et al., 2010). This is not surprising as previous studies have shown that peroxide production by planktonic *S. pneumoniae* elicits bactericidal effects on NTHi *in vitro* (Pericone et al., 2000). As recurrent OM is often considered a polymicrobial biofilm-associated disease, we performed studies to assess the relationship between these otopathogens in a dual species biofilm using our *in vitro* nasopharyngeal colonization model. Figure 1B shows that although NTHi can co-exist with *S. pneumoniae* for the initial 24 h, there is a rapid decline in NTHi viability to undetectable levels by 48 h. Based on these data, we concluded that NTHi and *S. pneumoniae* are unable to persist for extended periods in our *in vitro* model system.

### *Moraxella catarrhalis* and *S. pneumoniae* Form Dual Species Biofilms *in vitro*

To determine if the *S. pneumoniae* bactericidal activity versus NTHi was also elicited against *M. catarrhalis*, we assessed the compatibility of these bacteria in biofilms using our *in vitro* model system. In contrast to the NTHi data, *M. catarrhalis* viability in dual species biofilms with *S. pneumoniae* remained consistent with the monomicrobial biofilm control (Figure 1C). At 48 h both species were present in the biofilm at ∼10^6^ CFU per mL indicating that *M. catarrhalis* and *S. pneumoniae* can co-exist and persist in our model system *in vitro*. These data are consistent with previous *in vivo* studies suggesting that *M. catarrhalis* is capable of coping with the environmental stresses induced by *S. pneumoniae* in mixed species biofilms (Perez et al., 2014).

### *Moraxella catarrhalis* Promotes the Survival of NTHi in Polymicrobial Biofilms With *S. pneumoniae*

As chronic or recurrent OM is considered a biofilm-associated disease, we further assessed the ability of these three otopathogens to co-exist and persist in a polymicrobial biofilm. Figure 2 demonstrates that NTHi is now fully capable of surviving and persisting in this triple species biofilm even in the presence of *S. pneumoniae*. The survival of NTHi at 48 h was stastically significant as compared to NTHi in dual species with *S. pneumoniae* (Supplementary Figure S1). It appears that the presence of *M. catarrhalis* in these polymicrobial biofilms provides some form of protection from the bactericidal effects of *S. pneumoniae*. Further, at 48 h NTHi forms the most robust biofilms of all three otopathogens as quantified by CFU per mL.

Based on previous work done by Chao et al. (2017) we inoculated *S. pneumoniae* in all the previously mentioned time course assays at a lower seeding concentration to facilitate the analysis of bacterial-bacterial interactions. To ensure that our triple species data was not affected by lower seeding concentration, we completed a time course assay where all three otopathogens were diluted 1:100 and seeded simultaneously at approximately equivalent concentrations (∼10^4^ CFU per mL). Figure 3A confirms that the starting inocula had no effect on the establishment of polymicrobial biofilms and also confirms that the lower inoculum of *M. catarrhalis* was still able to protect NTHi from the bactericidal effects of the pneumococci.

### Multiple *M. catarrhalis* Clinical Isolates Protect NTHi in Polymicrobial Biofilms Containing *S. pneumoniae*

We further assessed the strain specificity of the protective effect by testing co-infection strains that were simultaneously isolated from the middle ear fluid of a pediatric patient during a middle ear infection. Data collected from the co-infection strains demonstrated that NTHi was able to persist within the triple species biofilm and that protection by *M. catarrhalis* is not strain specific (Figure 3B).

### Cell to Cell Contact Is Not Required to Protect NTHi From the Bactericidal Effects of *S. pneumoniae*

**FIGURE 4:**
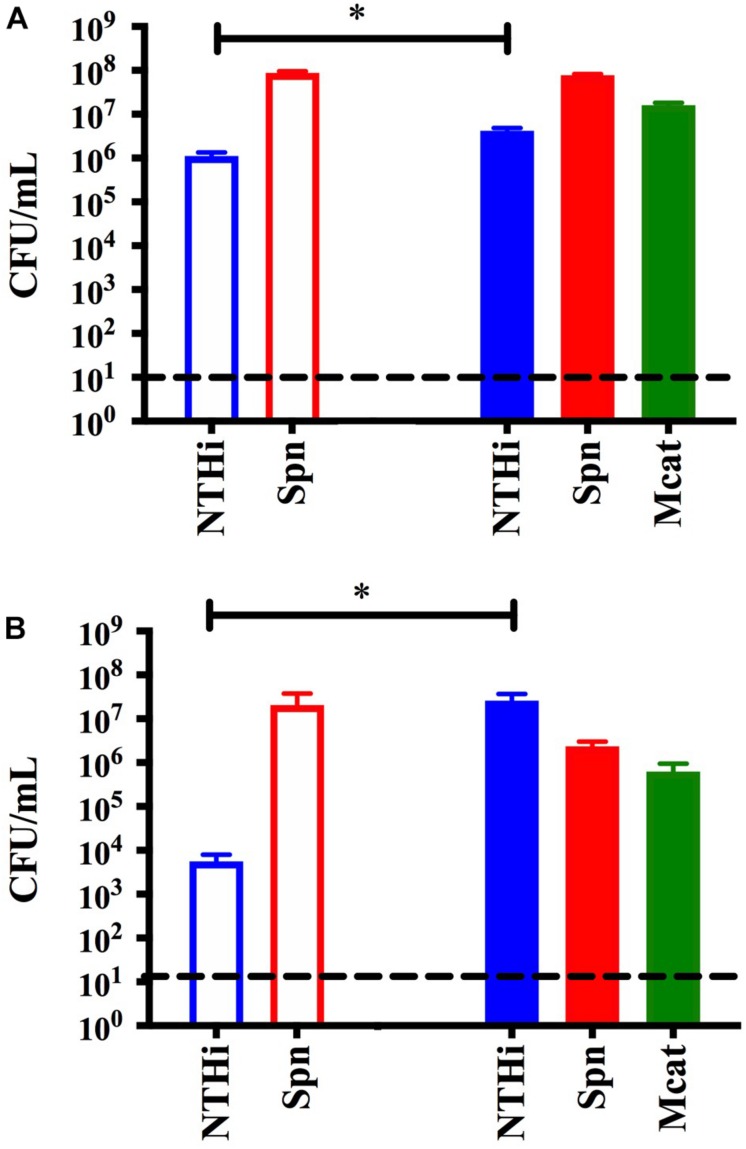
Transwell assay to assess protection without cell to cell contact. Dual species *S. pneumoniae* EF3030 (Spn) and NTHi 86-028 NP (NTHi) biofilms were seeded in 24-well plates on a fixed H292 cell monolayer. To the filter inserts, approximately 10^5^ CFU per mL *M. catarrhalis* 7169 (solid) or CDM (open) was added. Protection was assessed at **(A)** 24 h and **(B)** 48 h where bars represent the mean and SD of three independent assays with a minimum of four technical replicates. *P*-value of 0.05 was considered significant and indicated by asterisk (^∗^).

As one of the classic characteristics of bacterial biofilms is close contact at the cellular level, we utilized a transwell insert in our *in vitro* model system to physically isolate *M. catarrhalis* biofilms. Briefly, dual species *S. pneumoniae* and NTHi biofilms were seeded in 24-well plates on fixed H292 cell monolayers as described and transwell inserts were added to each well. *M. catarrhalis* was seeded into the inserts of the experimental wells and media alone was added to the control wells. Bacterial viability was assessed at 24 and 48 h as previously stated. Figure 4A shows that NTHi viability had a modest but significant decrease (∼1 log) at 24 h in the media treated controls as compared to the *M. catarrhalis* treated wells. This trend is even more apparent at 48 h, which shows that NTHi viability in the control wells was decreased by ∼10^3^ CFU/mL compared with the *M. catarrhalis* exposed dual species biofilms which actually showed an increase in NTHi bacterial persistence to ∼10^7^ CFU per mL (Figure 4B). These results demonstrate that *M. catarrhalis* can promote the survival and persistence of NTHi in the presence of *S. pneumoniae* and that this protective effect does not require direct contact. These data suggest that there is a factor(s) secreted or released into the supernatant that directly or indirectly confers protection to NTHi.

### *Moraxella catarrhalis* Catalase Production Is Not the Only Mechanism Involved in Protecting NTHi From Pneumococcal Hydrogen Peroxide

**FIGURE 5:**
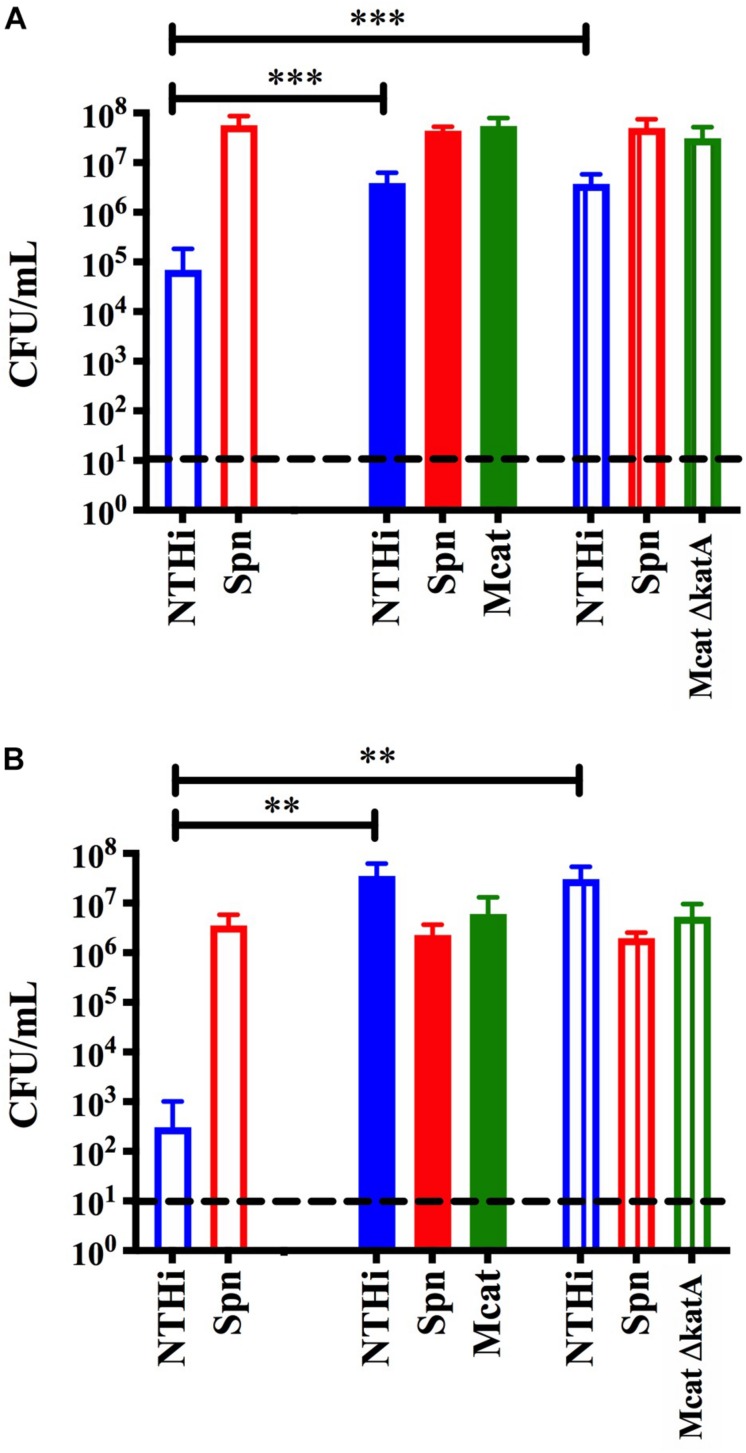
Transwell assay to assess the protective capacity of a *M. catarrhalis* catalase mutant. Dual species *S. pneumoniae* (SpnEF3030) and NTHi 86-028 NP (NTHi86028) biofilms were seeded in 24-well plates on a fixed H292 cell monolayer. To the transwell inserts, approximately 10^5^ CFU per mL *M. catarrhalis* 7169 (solid), Mcat7169 ΔkatASpec1 (vertical stripes) or CDM (open) was added. Protection was assessed at **(A)** 24 h and statistical analysis was performed using a one-way ANOVA and Dunnett’s multiple comparison test of log transformed CFU per mL **(B)** and 48 h and statistical analysis was performed on log transformed CFU per mL using the Kruskal–Wallis with Dunn’s multiple comparison test. Bars represent the mean and standard deviation of three independent assays with a minimum of two technical replicates. *P*-value of <0.01 denoted ^∗∗^ and *P*-value of <0.001 denoted ^∗∗∗^.

Previous studies have shown that the bactericidal effects of *S. pneumoniae* hydrogen peroxide production on NTHi can be reversed by the addition of catalase (Pericone et al., 2000). Although NTHi produces catalase, the activity *in vitro* is considered quite low (Pericone et al., 2000) and this likely explains why it is not protective in our model system (Figure 1B). However, *M. catarrhalis* does produce a highly active catalase and this has been previously shown to neutralize the effects of *S. pneumoniae* hydrogen peroxide in planktonic cultures *in vitro* (Pericone et al., 2000). Based on this previous report, we constructed an isogenic catalase mutant in the *M. catarrhalis* 7169 (Mcat7169ΔkatASpec1) and confirmed this mutant was defective in catalase production (data not shown). This Mcat7169ΔkatASpec1 construct was assessed for protective activity in the transwell assay versus the wild-type Mcat7169 at 24 h (Figure 5A) and 48 h (Figure 5B). These results show that the Mcat7169ΔkatASpec1 mutant was able to confer protection of NTHi to the same extent as the wild type Mcat7169 at both time points. Given the previous reports, these results were somewhat surprising suggesting that catalase is not solely responsible for the protective effect seen in these polymicrobial biofilms using our model system.

## Discussion

The current model for OM pathogenesis is based on the concept that commensal bacteria colonize the nasopharynx of children very early in life and provide a reservoir for invasive diseases including AOM, sinusitis, and bacterial pneumonia. Although the mechanism of transition from colonization to opportunistic infection is not fully understood, it is widely accepted that changes in the host during a viral upper respiratory infection are linked to the subsequent onset of AOM (Heikkinen and Chonmaitree, 2003; Chonmaitree et al., 2008, 2016; Nokso-Koivisto et al., 2015). Based on this model, biofilms associated with colonization of otopathogens in the nasopharynx play a fundamental role in pathogenesis and are thus a primary target for preventative strategies.

While little is known about the transition from asymptomatic colonization to active infection, even less is known about how polymicrobial communities play a role in this transition. Using a model designed to mimic the environment of the nasopharynx we assessed the three primary otopathogens in dual species and triple species biofilms *in vitro*. To date, some of the dual species interactions have been characterized, but with contrasting results. For example, *S. pneumoniae* hydrogen peroxide production has been shown to have bactericidal effects on the other respiratory tract pathogens in planktonic cultures, in addition to being cytotoxic to the respiratory epithelial cells (Pericone et al., 2000). Likewise, *S. pneumoniae* produces neuraminidase which has the potential to remove sialic acid residues from other bacterial species exposing them to the host immune system. However, despite the many *S. pneumoniae* virulence factors cited, Perez et al. (2014) reported that *S. pneumoniae* increased colonization of *M. catarrhalis in vivo*. The same study also demonstrated that *M. catarrhalis* is able to confer β-lactamase protection to *S. pneumoniae* and conversely *S. pneumoniae* is able to provide *M. catarrhalis* with macrolide resistance. Based on the current literature, many questions remain relating to the potential synergism or antagonistic co-colonization of these two respiratory pathogens. The data presented in this study confirm that *M. catarrhalis* is in fact able to co-colonize in dual species biofilms with *S. pneumoniae in vitro*.

The multivalent pneumococcal conjugate vaccine and the *Haemophilus influenzae* type b vaccine have changed the landscape of nasopharyngeal colonization (Cohen et al., 2006; Revai et al., 2006; Dagan, 2009). Despite the success of these vaccines, NTHi strains and non-vaccine serotypes of *S. pneumoniae* remain a prominent cause of AOM. One of the few studies on the interactions of *S. pneumoniae* and NTHi suggests that NTHi can also confer β-lactamase-dependent antibiotic resistance and promote biofilm formation, and thus increase persistence, *in vivo* (Weimer et al., 2010, 2011). When studied *in vitro*, the interactions between *S. pneumoniae* and NTHi were reported to be cell density dependent and required the down regulation of pneumococcal genes regulating autolysis and fratricide (Hong et al., 2014). Our results indicate that *S. pneumoniae* is bactericidal to NTHi in dual species biofilms despite seeding at a relatively low concentration of *S. pneumoniae* to limit cytotoxicity. After approximately 24 h of biofilm growth, NTHi began to decline as a result of *S. pneumoniae* virulence.

We were also able to confirm that *M. catarrhalis* and NTHi successfully co-colonize in dual species biofilms as previously reported (Armbruster et al., 2010). Additionally, in our dual species model *M. catarrhalis* is able to promote the biofilm formation of NTHi supporting recently published work by Mokrzan et al. (2018). These observations have important implications because prolonged nasopharyngeal carriage subsequently increases the potential for transition to disease.

The nasopharynx is a polymicrobial environment. Multiple studies have shown that *S. pneumoniae*, *M. catarrhalis* and NTHi can simultaneously occupy this mucosal niche; however, very little is known about the bacterial interactions that occur between these species and the possible role these play in the pathogenesis of AOM (Hoa et al., 2009; Casey et al., 2010; Palmu et al., 2019). As mentioned previously, studies have shown that *M. catarrhalis* is more likely to be found in polymicrobial AOM infections than from a single-species infection (Broides et al., 2009). Additionally, colonization with *M. catarrhalis* happens very early in life, which is in part why *M. catarrhalis* is more frequently seen in children experiencing their first AOM episode as opposed to those with recurrent infections (Broides et al., 2009). These two qualities suggest that *M. catarrhalis* may be one of the first bacteria-bacteria interactions and a major factor in the subsequent colonization of other bacterial species. To test the possibility that *M. catarrhalis* may be important in polymicrobial colonization, we analyzed all three bacterial species in our colonization model *in vitro*. Our data confirmed that all three otopathogens were able to form a mixed species biofilm in our model system. More importantly, the presence of *M. catarrhalis* was essential for NTHi to survive the bactericidal effects of *S. pneumoniae*. This phenomenon, which appears to be “protective” for NTHi, has not been documented to date and provides novel insight into nasopharyngeal co-colonization and subsequently polymicrobial infection pathogenesis.

To explore the mechanism of protection we first considered the physical properties of the biofilms. We addressed the possibility that biofilm architecture was involved in shielding NTHi from *S. pneumoniae* or that cell to cell contact was required. We utilized a transwell assay which physically separated *M. catarrhalis* biofilms from NTHi and *S. pneumoniae* dual species biofilms, but allowed for the exchange of components in the supernatant. We found that physical contact is not necessary for *M. catarrhalis* to confer protection. These data suggest that a secreted or released factor(s) is responsible for the observed protective effect. We considered the known bactericidal effects of *S. pneumoniae* hydrogen peroxide production and hypothesized that catalase production may play a role in this process (Pericone et al., 2000). To test this, we constructed a catalase mutant in the *M. catarrhalis* 7169 background (Mcat7169ΔkatASpec1) and assessed its protective capacity in the transwell assay. The mutant had a phenotype similar to the wild-type demonstrating that catalase production by *M. catarrhalis* was not essential for protection. It is possible that Mcat7169ΔkatASpec1 was able to compensate using other mechanisms for coping with oxidative stress. Our initial results do not indicate a clear mechanism of protection, but they do suggest that it is reliant on a secreted or released factor(s) that is not catalase and is not dependent on physical or architectural characteristics of polymicrobial biofilms.

Future studies aim to use a more global approach to analyze the transcriptome of *M. catarrhalis* monomicrobial biofilms versus *M. catarrhalis* polymicrobial biofilms to parse out genes that are important in the polymicrobial environment and thus potentially important for the protective effect. The cumulative ability of these otopathogens to form polymicrobial biofilms, persist, and resist antibiotics has major implications in AOM epidemiology and pathogenesis. The mechanism of protection could provide a target for minimizing polymicrobial biofilms of the nasopharynx.

## Data Availability Statement

The raw data supporting the conclusions of this article will be made available by the authors, without undue reservation, to any qualified researcher.

## Author Contributions

KB performed all the aforementioned experimentation. KB and AC contributed to the conception of the experimental design and preparation of the manuscript.

## Conflict of Interest

The authors declare that the research was conducted in the absence of any commercial or financial relationships that could be construed as a potential conflict of interest.

## Supplementary Material

FIGURE S1 A 48 h survival assessment. Bacterial species were statistically analyzed under each polymicrobial growth condition as compared to the monomicrobial control. Bars represent the mean and SD of minimally three independent assays. **(A)** NTHi, **(B)** *S. pneumoniae*, and **(C)** *M. catarrhalis* growth conditions were log transformed and analysis was completed using a student’s *t*-test or a Mann–Whitney test based on the Shapiro–Wilk test for normality. *P*-value of <0.05 denoted ^∗^, <0.01 denoted ^∗∗^, and <0.001 denoted ^∗∗∗^.
