# Supplementary figures and images for: Moraxella catarrhalis Promotes Stable Polymicrobial Biofilms With the Major Otopathogens
Source: Front Microbiol. 2020 Jan 15;10:3006. doi: 10.3389/fmicb.2019.03006 (PMC6974515; doi:10.3389/fmicb.2019.03006)

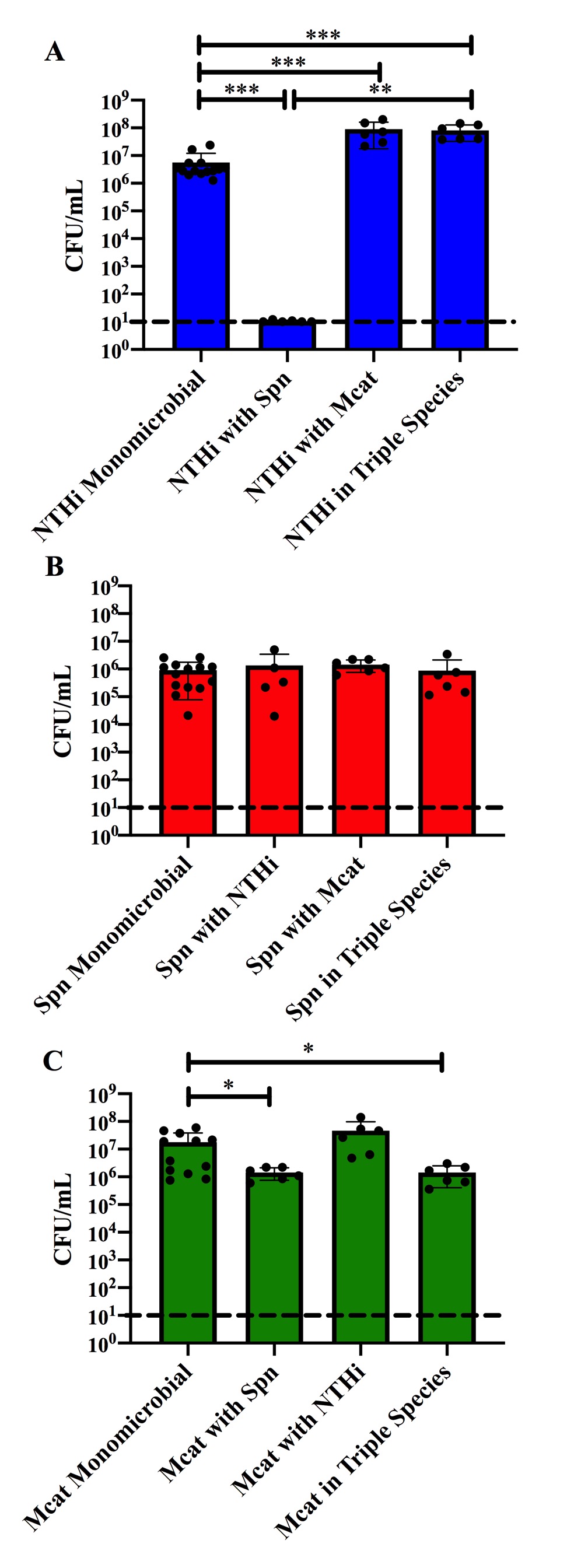

Supplement: FIGURE S1 — A 48 h survival assessment. Bacterial species were statistically analyzed under each polymicrobial growth condition as compared to the monomicrobial control. Bars represent the mean and SD of minimally three independent assays. (A) NTHi, (B) S. pneumoniae, and (C) M. catarrhalis growth conditions were log transformed and analysis was completed using a student’s t-test or a Mann–Whitney test based on the Shapiro–Wilk test for normality. P-value of <0.05 denoted ∗, <0.01 denoted ∗∗, and <0.001 denoted ∗∗∗. [file Image_1.JPEG]
